# Impact of expressive suppression on subthreshold depression among college students: a moderated mediation model

**DOI:** 10.3389/fpsyg.2025.1678275

**Published:** 2025-10-22

**Authors:** Mingxuan Chen, Lieyu Huang, Qian Qin, Jinlu Li, Jiarui Zhang, Jing Ma, Haoyang Song

**Affiliations:** ^1^Department of Medical Humanities, Guizhou Medical University, Guiyang, China; ^2^Department of Nursing, Jiangxi Medical College, Shangrao, China; ^3^Department of Mental Health, Shangrao Normal College, Shangrao, China; ^4^Department of Psychiatry, Affiliated Hospital of Zunyi Medical University, Zunyi, China

**Keywords:** subthreshold depression, expressive suppression, regulatory emotional self-efficacy, negative attention bias, college students

## Abstract

**Introduction:**

Emotion regulation strategies, particularly expressive suppression, and negative attention bias significantly contribute to the onset and persistence of subthreshold depression among college students. However, the underlying moderating mechanisms of this relationship remain inadequately understood. Guided by the Emotion Regulation Process Model, this study examined a moderated mediation model to determine whether negative attention bias mediates the relationship between expressive suppression and subthreshold depression and to explore the moderating role of regulatory emotional self-efficacy within this framework.

**Methods:**

A total of 956 college students aged between 19 and 24 years participated in this cross-sectional survey.

**Results:**

Negative attention bias fully mediated the relationship between expressive suppression and subthreshold depression. Moreover, regulatory emotional self-efficacy moderated both the direct relationship between expressive suppression and subthreshold depression, and the association between negative attention bias and subthreshold depression. Specifically, individuals with higher levels of regulatory emotional self-efficacy exhibited diminished indirect effects of expressive suppression on subthreshold depression through negative attention bias.

**Discussion:**

These findings provide valuable insights into potential mechanisms for targeted interventions and preventative measures aimed at reducing depressive symptoms among college students.

## 1 Introduction

Depression is a prevalent mental health disorder characterized by core symptoms such as persistent low mood, anhedonia, sleep disturbances, cognitive impairments, and diminished energy (Beck et al., 1996; Paykel, 2008; Stringaris, 2017; Shorey et al., 2022). According to the World Health Organization (World Health Organization [WHO], 2023), approximately 280 million individuals globally suffer from depression, and annually over 700,000 suicides are associated with depression, making suicide the fourth leading cause of death among individuals aged 15–29 (Thapar et al., 2022; Grossberg and Rice, 2023). Compared with clinical depression, subthreshold depression features milder symptoms and shorter durations, yet significantly impacts psychological functioning, social adaptation, and overall quality of life (Jiao et al., 2025; Volz et al., 2023; Ge et al., 2024). Empirical evidence indicates that college students constitute a particularly high-risk group for subthreshold depression (Ling et al., 2021; Langer et al., 2022), with prevalence rates notably higher than in the general adult population (Auerbach et al., 2018; Beiter et al., 2015). In China, a large-scale survey involving approximately 80,000 college students reported a subthreshold depression prevalence rate of 21.48% (Mofatteh, 2020). Similarly, a meta-analysis of 84 studies covering 1,292,811 college students estimated this prevalence at 26% (Luo et al., 2021). These findings highlight the substantial burden of depression among college students, impairing their physical and mental health, disrupting academic performance, weakening interpersonal relationships, and significantly increasing suicide risk (Hammen, 2018). Therefore, identifying underlying mechanisms of subthreshold depression among college students is imperative for developing targeted interventions and preventive measures.

Emotional dysregulation is a core characteristic of depression, making emotion regulation strategies a critical intervention target (Beck, 2008; Beck et al., 2021). Gross’s process model of emotion regulation emphasizes cognitive reappraisal and expressive suppression (ES) as primary regulatory strategies (Gross et al., 1997; Gross and John, 2003). CR, an antecedent-focused strategy, effectively mitigates negative affect, whereas ES, a response-focused strategy, may temporarily inhibit emotional expression but potentially exacerbates depressive symptoms when overused (Troy et al., 2023; Gross and Cassidy, 2024). Although attentional bias toward negative stimuli has been suggested as a potential mediator between emotional regulation strategies and depression, this mechanism remains largely unexplored and lacks empirical validation in the context of expressive suppression and subthreshold depression. Furthermore, the moderating role of regulatory emotional self-efficacy (RESE) in this context lacks robust empirical validation. Integrating these perspectives, this study employs the emotion regulation process model to investigate how negative attentional bias mediates the relationship between ES and subthreshold depression, and how RESE moderates these associations, thus elucidating pathways from maladaptive emotion regulation strategies to subthreshold depression.

### 1.1 Expressive suppression and subthreshold depression

Expressive suppression, defined as the deliberate inhibition of overt emotional expressions post-emotional activation (Gross, 1998; Gross and Cassidy, 2019), operates late in the emotional response process without modifying the emotional experience itself. Chronic reliance on ES can intensify emotional dysregulation by increasing physiological arousal and promoting cognitive rumination, thereby exacerbating depressive symptoms (Aldao et al., 2010; Gross and John, 2003; Li et al., 2019). Empirical evidence consistently demonstrates that habitual ES is associated with adverse mental health outcomes, including depressive symptoms (Chavez-Baldini et al., 2020; Solak et al., 2021; Van Eickels et al., 2022; Cui et al., 2024; Qu et al., 2024; Li Y. et al., 2024; Thuillard and Dan-Glauser, 2020; Anderson et al., 2021). A meta-analysis by Liu et al. (2020) comprising 106 studies in China further confirmed the positive correlation between ES and depression. Additionally, Blalock and Reyna (2016) observed higher ES levels among socially anxious college students, correlating strongly with depressive symptoms. Despite the evidence linking ES to subthreshold depression, the underlying mechanisms, particularly the mediating roles of attentional biases and RESE, require further empirical clarification.

### 1.2 Negative attentional bias as a mediator

Negative attentional bias indicates the tendency to automatically focus on negative emotional stimuli during cognitive processing (Gross, 1998, 2015). Chronic use of ES tends to foster biased attention toward negative stimuli, resulting in sustained negative affect and heightened depressive symptoms (Yan et al., 2022; Liu et al., 2023; Huang et al., 2023; Gou et al., 2023). Empirical research suggests negative attentional bias may mediate the relationship between ES and subthreshold depression, although existing studies primarily rely on correlational analyses (Gupta et al., 2019; Zhang, 2023; Stellern et al., 2023; Liu et al., 2024; Patrichi et al., 2025; Özdemir and Yüksel, 2025). When individuals suppress their emotional expressions, their attention becomes more focused on negative stimuli, which exacerbates negative mood and depressive symptoms. Unlike other cognitive processes, attentional bias operates at an early stage of cognitive processing, making it a proximal and direct link between emotional regulation strategies and depressive symptoms. Beck (2008) emphasized the robust association between negative attentional bias and depressive symptoms, further supported by meta-analytic evidence highlighting the role of cognitive biases in disrupting emotion regulation and perpetuating depressive states (Xia et al., 2023). Nevertheless, systematic validation of negative attentional bias as a mediator between ES and subthreshold depression among college students remains limited, warranting further investigation.

### 1.3 Regulatory emotional self-efficacy as a moderator

Regulatory emotional self-efficacy is the subjective confidence in effectively managing emotional experiences (Caprara et al., 2005). RESE has been shown to play a crucial role in moderating the relationship between emotion regulation strategies, such as ES, and mental health outcomes, including depression. Emerging evidence suggests RESE moderates the indirect relationship between ES and depression mediated by negative attentional bias (Bardeen and Fergus, 2020; Li S. et al., 2024). According to Bandura’s self-efficacy theory (Bandura et al., 2003), individuals with higher RESE tend to use adaptive cognitive strategies to regulate negative emotions, while those with lower RESE face greater difficulty disengaging from negative stimuli post-ES, thus exacerbating depressive symptoms (Visted et al., 2018). Cross-sectional and longitudinal studies have demonstrated RESE’s protective effect against depression and anxiety (Mesurado et al., 2018; Zhang et al., 2022; Lu et al., 2025). RESE, therefore, functions as an adaptive coping resource that is particularly activated when individuals are already confronted with negative cognitive biases, such as the formation of negative attentional bias. This protective effect is more pronounced in the later stages of the emotional regulation process, after negative attentional bias has already been established, rather than during the initial selection of emotion regulation strategies (e.g., expressive suppression). High RESE enables individuals to effectively cope with and reframe their cognitive biases, thereby reducing the risk of depression. In contrast, low RESE limits the individual’ s ability to regulate their emotional reactions once negative attentional bias has been triggered, leading to the exacerbation of depressive symptoms.

### 1.4 The present study

**FIGURE 1:**
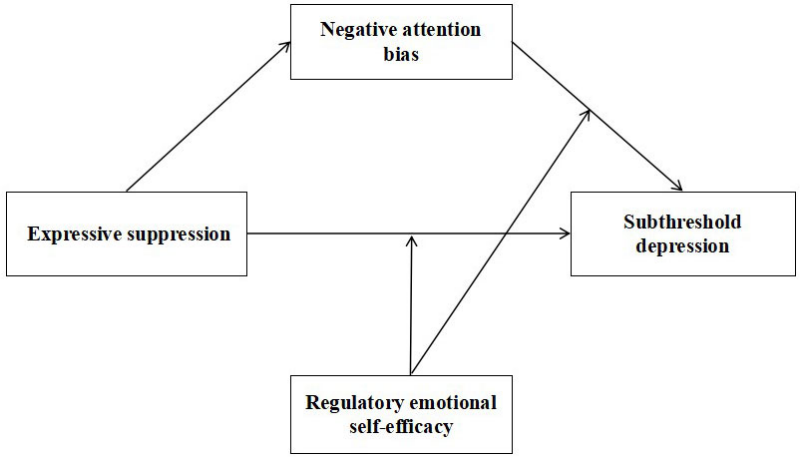
The hypothesized moderated mediation model.

Grounded in the emotion regulation process model, this study investigates the moderated mediation model involving ES, negative attentional bias, and subthreshold depression, moderated by RESE among college students (Figure 1). Specifically, we hypothesize:

Hypothesis 1: Expressive suppression will significantly predict subthreshold depression.

Hypothesis 2: Negative attentional bias mediates the relationship between ES and subthreshold depression.

Hypothesis 3: RESE moderates the indirect relationship between ES and subthreshold depression through negative attentional bias, such that the mediation effect is stronger for individuals with lower RESE compared to those with higher RESE.

## 2 Materials and methods

### 2.1 Participants

Participants and Procedures Participants were recruited using a cluster convenience sampling method from a university located in Western China. Initially, 990 undergraduate students consented to participate and completed the survey. Following exclusion of 34 incomplete responses, the final sample comprised 956 valid questionnaires, yielding a response rate of 96.57%. The exclusion criteria for incomplete responses were clearly defined as follows: (1) participants who failed the lie detection questions (designed to identify invalid or careless responses) and (2) participants with more than 20% missing data. Participants ranged in age from 19 to 24 years, with a mean age of 19.61 years (SD = 1.11). Males represented 58.60% of the sample. Ethical approval for the study protocol was granted by the institutional ethics committee, and all participants provided informed consent prior to data collection.

### 2.2 Materials

#### 2.2.1 Depression

Depressive symptoms were assessed using the Beck Depression Inventory-II (BDI-II; Beck et al., 1996), one of the most extensively validated self-report measures of depression. The BDI-II comprises 21 items evaluating various dimensions, including emotional state, loss of interest, sleep disturbances, energy levels, and suicidal ideation. Respondents rate each item on a four-point Likert scale, resulting in total scores ranging from 0 to 63, with higher scores indicative of greater depressive symptom severity. The BDI-II has previously demonstrated satisfactory reliability and validity among Chinese college students (Yang et al., 2012). In the current study, Cronbach’s alpha was 0.92.

#### 2.2.2 Negative attentional bias

Negative attentional bias was measured using the Negative Attentional Bias subscale from the Attentional Bias Scale developed by Noguchi et al. (2006), which was subsequently adapted for Chinese adolescents by Lü et al. (2016). The original scale contains 30 items divided into positive (19 items) and negative (11 items) attentional bias subscales. The Negative Attentional Bias subscale assesses the tendency to preferentially focus on negative information (e.g., “I worry that bad things will happen to me”). Participants rated each item on a five-point Likert scale ranging from 1 (completely inconsistent) to 5 (completely consistent) based on their experiences over the past 1–2 weeks. In the present study, the Cronbach’s alpha for the Negative Attentional Bias subscale was 0.83.

#### 2.2.3 Expressive suppression

Expressive suppression was assessed using the Expressive Suppression subscale from the Emotion Regulation Questionnaire (ERQ; Gross and John, 2003). This widely-used instrument evaluates the frequency of individuals’ use of specific emotion regulation strategies. The ERQ consists of 10 items, with the Expressive Suppression subscale comprising four items (e.g., “I control my emotions by not expressing them”). Items are rated on a seven-point Likert scale from 1 (strongly disagree) to 7 (strongly agree). The ERQ has demonstrated satisfactory psychometric properties in Chinese populations (Wang et al., 2007). The Expressive Suppression subscale in this study had a Cronbach’s alpha of 0.72.

#### 2.2.4 Regulatory emotional self-efficacy

Regulatory emotional self-efficacy was assessed using the Regulatory Emotional Self-Efficacy Scale (RESES; Caprara et al., 2005), adapted for Chinese populations by Zhang et al. (2010). The RESES comprises 12 items evaluating two domains: self-efficacy in expressing positive emotions (e.g., “I can share my joy with others”) and self-efficacy in regulating negative emotions (e.g., “I can calm down quickly when I am angry”). Participants rated their confidence level in regulating emotions on a five-point Likert scale ranging from 1 (strongly disagree) to 5 (strongly agree). Higher scores indicate stronger perceived emotional self-efficacy. Cronbach’s alpha for the total scale in the current study was 0.81.

### 2.3 Data analysis

Data analysis was performed using SPSS version 26.0. First, descriptive statistics including means, standard deviations, correlations, and reliability coefficients for each measure were computed. Prior to conducting analyses, we assessed the model assumptions. The assumption of linearity was confirmed through scatterplots, which showed a linear relationship between the independent and dependent variables. Furthermore, we assessed multicollinearity by calculating the variance inflation factors (VIFs) for all predictor variables in the models. All VIF values were well below the conservative threshold of 5, indicating that multicollinearity was not a concern. Missing data were handled using maximum likelihood estimation (MLE), which is considered an efficient method for dealing with missing data under the assumption that data are missing at random (MAR). Subsequently, we tested the proposed mediation model using Model 4 of the PROCESS macro for SPSS (Hayes, 2013) to examine indirect effects. To further investigate whether regulatory emotional self-efficacy moderated both direct and mediated relationships, a moderated mediation analysis was conducted using Model 15 of the PROCESS macro. Prior to analyses, all continuous variables were standardized. Gender was entered as a covariate in all analyses due to its documented potential influence on depressive symptoms (Nolen-Hoeksema and Aldao, 2011). Also, preliminary analyses also confirmed the presence of a significant gender difference in levels of subthreshold depression. To assess the potential influence of common method bias, we conducted Harman’ s single-factor test. The results showed that ten factors had eigenvalues greater than 1, and the first factor accounted for 22% of the variance, which is below the critical threshold of 40%. These results suggest that common method bias was not a serious concern in this study.

## 3 Results

### 3.1 Descriptive statistics

Table 1 presents the descriptive statistics and intercorrelations between variables. ES, negative attentional bias, RESE, and Depression were significantly correlated with each other (−0.46 < r < −0.09, 0.16 < r < 0.42, *p* < 0.01).

**TABLE 1:** Descriptive statistics and intercorrelations between variables.

Variables	M	SD	1	2	3	4
Depression	7.82	8.07	–	–	–	–
ES	15.37	3.70	0.16***	–	–	–
Negative attentional bias	34.38	5.66	0.42***	0.31***	–	–
RESE	42.46	5.54	−0.46***	−0.09**	−0.32***	–

*p* < 0.05;**p < 0.01;***p < 0.001; *n* = 956; ES, expressive suppression; RESE, regulatory emotional self-efficacy.

### 3.2 Testing for mediation effect

We employed Model 4 of the PROCESS macro for SPSS to test the mediation effect, with results presented in Table 2. Controlling for gender, ES significantly predicted negative attentional bias (β = 0.35, *p* < 0.001), indicating that higher levels of ES were associated with greater negative attentional bias in students. Negative attentional bias significantly predicted depression (β = 0.42, *p* < 0.001), suggesting that students who focus more on negative stimuli tend to have higher levels of depression. The total effect of expressive suppression on depression was also significant [total effect = 0.18, *p* < 0.001, 95% CI (0.11, 0.25)]. However, the residual direct effect of expressive suppression on depression was not statistically significant (β = 0.03, *p* > 0.05), indicating that after accounting for negative attentional bias, ES did not have a direct impact on depression. Thus, Hypothesis 1 is not supported. Additionally, the indirect effect of expressive suppression on depression via negative attentional bias was significant [indirect effect = 0.15, 95% CI (0.11, 0.18)], confirming Hypothesis 2, which means that the relationship between ES and depression is mediated through negative attentional bias.

**TABLE 2:** Summary table of mediation effect analysis.

Predictors variables	Negative attentional bias		Depression	
	β	*t*	β	*t*
Gender	0.17**	2.81	0.01	−0.05
ES	0.35***	10.40	0.04	1.02
Negative attentional bias	–	–	0.42***	13.25
R^2^	0.10		0.18	
F	54.71***		68.82***	

*p* < 0.05;**p < 0.01;***p < 0.001; *n* = 956; ES, expressive suppression; RESE, regulatory emotional self-efficacy.

### 3.3 Testing for moderated mediation

The moderating effects of ERSE (Hypothesis 3) on the mediation model were tested using Model 15 of the PROCESS macro (Hayes, 2013). Results for each moderated mediation model are presented in Table 3. As can be seen in Table 3, a significant two-way interaction between ES and RESE emerged (β = −0.07, *p* < 0.05), suggesting that RESE moderated the direct relationship between ES and depression. Moreover, another significant two-way interaction between negative attentional bias and ERSE predicting depression was also found (β = −0.06, *p* < 0.05). This suggests that RESE also moderated the relationship between negative attentional bias and depression.

**TABLE 3:** Testing the moderated mediation effects of emotion regulation self-efficacy between emotional inhibition expression and depression.

Predictors variables	Model 1 negative attentional bias		Model 2 depression	
	β	*t*	β	*t*
Gender	0.170**	2.81	−0.09	−1.62
ES	0.35***	10.40	0.03	1.06
RESE	–	–	−0.39***	−12.85
ES × RESE	–	–	−0.07*	−2.19
Negative attentional bias	–	–	0.31***	10.22
Negative attentional bias × RESE	–	–	−0.06*	−2.51
R^2^	0.10		0.31	
F	54.71***		70.71	

*p < 0.05;**p < 0.01;***p < 0.001; *n* = 956; ES, expressive suppression; RESE, regulatory emotional self-efficacy.

**FIGURE 2:**
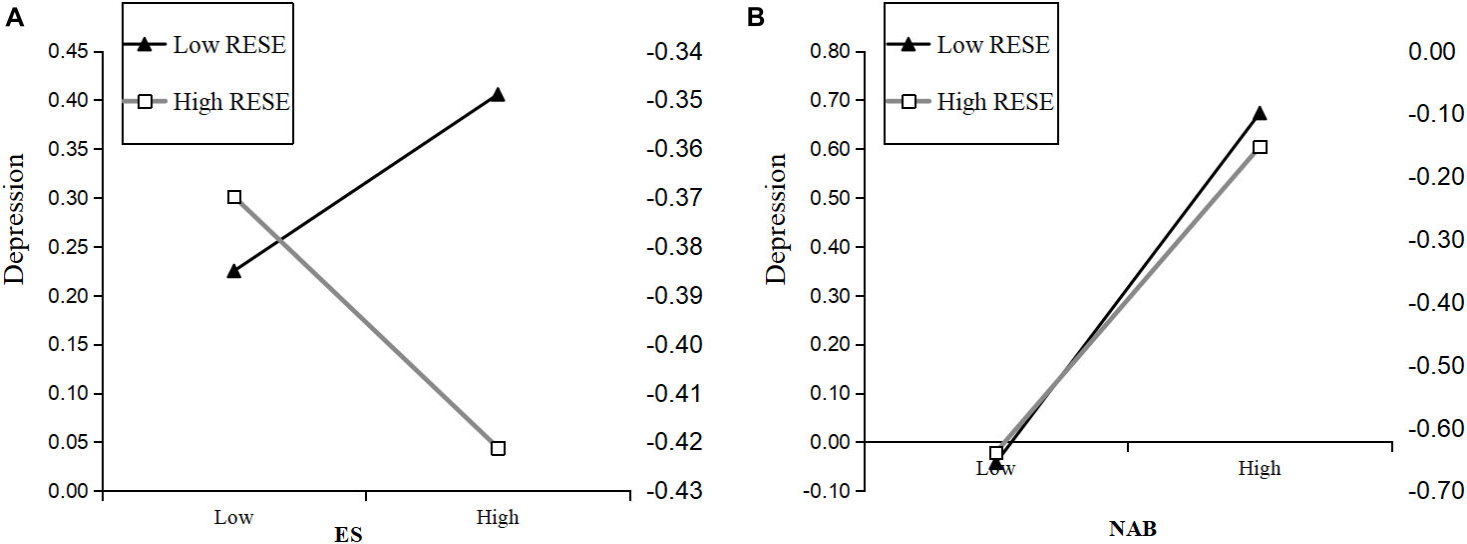
**(A)** Simple slope of the association between ES and depression at low vs. high levels of RESE. **(B)** Simple slope of the association between NAB and depression at low vs. high levels of RESE; ES, expressive suppression; NAB, negative attentional bias; RESE, regulatory emotional self-efficacy.

To further examine the interaction between ES and depression, the relationship between the two variables was plotted across two levels of RESE (i.e., 1 standard deviation below the mean and 1 standard deviation above the mean). As illustrated in Figure 2A, when RESE was low, the impact of ES on depression was statistically significant (β = 0.10, *p* < 0.01). However, this was not the case for participants with high RESE (β = −0.03, *p* > 0.05). This suggests that higher RESE may buffer the negative effects of expressive suppression on depression. Also, as illustrated in Figure 2B, for individuals with low RESE, the effect of negative attentional bias on depression was significant (β = 0.38, *p* < 0.001), this association became weaker among individuals with high RESE (β = 0.26, *p* < 0.001). This indicates that RESE moderated the indirect effect of ES on depression through negative attentional bias, suggesting that individuals with higher RESE are less susceptible to the negative effects of attentional bias in the relationship between ES and depression.

## 4 Discussion

Contrary to Hypothesis 1, the direct effect of expressive suppression on subthreshold depression was not statistically significant after controlling for negative attentional bias. This finding does not align with Liu et al. (2020), who found a significant positive correlation between expressive suppression and negative mental health outcomes, including depression. One possible explanation for the non-significant direct effect is that expressive suppression may not have a uniform or immediate impact on depressive symptoms across individuals. Instead, its effect may depend on how it alters cognitive mechanisms, such as attention, memory, or interpretation bias. Another potential reason lies in the nature of subthreshold depression itself. Compared to clinical depression, subthreshold depression may be more sensitive to cognitive rather than emotional dysregulation directly. That is, individuals with subthreshold symptoms might not experience the immediate emotional consequences of suppression, but their habitual use of suppression may gradually bias their attention toward negative stimuli, eventually contributing to mood deterioration. Statistically, the lack of a significant direct effect emphasizes the complexity of the relationship between expressive suppression and depression, suggesting that its effects may be more indirect and mediated by cognitive biases. Clinically, the results imply that interventions focused solely on expressive suppression may be insufficient for individuals with subthreshold depression. Instead, addressing cognitive biases, such as negative attentional bias, could be more effective in preventing or mitigating depressive symptoms.

Aligned with previous findings (Xu et al., 2015; Xiao et al., 2022; Li S. et al., 2024; Faul et al., 2024), this study supports Hypothesis 2 by confirming negative attentional bias as a significant mediator between ES and subthreshold depression. While ES suppresses external emotional displays, it does not mitigate internal negative affect; rather, it amplifies selective attention toward negative stimuli, reinforcing negative attentional bias. This heightened attentional bias toward negative events (e.g., academic failures, social conflicts) facilitates their detection and prolonged cognitive processing (Wu et al., 2022), activating maladaptive cognitive schemas, reducing emotional regulation flexibility, and accelerating negative affect accumulation among college students. These findings illustrate a “suppression–attentional bias–depression” cycle that contributes to depressive symptomatology.

Furthermore, our results revealed that RESE moderated both the direct effect of ES on subthreshold depression and the indirect effect mediated by negative attentional bias. Although the effect sizes for the moderation (β = −0.07 and −0.06) are small, they are statistically significant, which emphasizes the importance of RESE as a protective factor in emotion regulation. Even small effect sizes can have significant clinical implications, particularly in large populations or over extended periods. This dual moderating effect implies that higher RESE can buffer the detrimental consequences of ES. Individuals with high RESE more effectively employ compensatory strategies (e.g., mindfulness, positive reappraisal) to alleviate emotional distress, whereas those with low RESE exhibit stronger associations between ES and depression. These findings align with Bandura’s (1997), Bandura et al., 2003) assertion that belief in one’s emotional regulation abilities can mitigate the adverse effects of maladaptive strategies. Additionally, the moderating role of RESE in the negative attentional bias-depression relationship further highlights its protective function across cognitive and emotional domains, enhancing psychological resilience and attenuating emotional deterioration stemming from maladaptive cognitive processing.

Several limitations must be acknowledged. First, the cross-sectional design limits causal inferences and precludes an examination of potential reciprocal or temporal dynamics between ES and subthreshold depression. This design does not allow us to assess whether the effect of expressive suppression on depression is bidirectional, nor does it capture whether the attentional bias amplifies the effects of suppression over time. Although cross-sectional data provides valuable insights into the associations between these variables, a longitudinal design is needed to establish causal relationships and explore the dynamic pathway, such as whether negative attentional bias increases the impact of expressive suppression on subthreshold depression over time. Future research employing panel data could validate these pathways and further illuminate the temporal dynamics underlying the relationship between emotion regulation and depressive symptoms. Second, the use of cluster convenience sampling from a single university in Western China limits the external validity of our findings. The generalizability may be influenced by several regional and sociodemographic characteristics specific to this context. For instance, socioeconomic disparities, cultural norms regarding emotional expression prevalent in Western China, and the particular academic stressors faced by students in this region may differ from those in more developed Eastern Chinese cities or other cultural settings. These factors could potentially alter the dynamics between expressive suppression, negative attentional bias, and subthreshold depression. Therefore, future research should strive to replicate this study using more representative, nationwide samples that encompass a wider range of universities from various geographic and socioeconomic backgrounds. Third, reliance on self-report measures introduces potential biases, such as symptom underreporting or inflated self-efficacy estimations. Future studies should employ multimethod approaches, incorporating behavioral and physiological assessments (e.g., eye-tracking to measure attentional biases). Lastly, while this research focused on negative attentional bias and RESE, other potential mediators (e.g., rumination) and moderators (e.g., social support) merit further exploration. Specifically, rumination has been shown to exacerbate negative thinking patterns and plays a significant role in the development and persistence of depression (Moulds et al., 2022). Future studies could examine how rumination might mediate the relationship between emotional regulation strategies and depression. Similarly, social support has been identified as a protective factor that buffers the detrimental effects of emotional dysregulation and may serve as an important moderator in the relationship between expressive suppression and depression (Gariépy et al., 2016). These variables were not included in the current study due to scope limitations, but future research could incorporate them to provide a more comprehensive understanding of the mechanisms at play and refine interventions aimed at reducing depressive symptoms.

Despite these limitations, the current findings hold substantial theoretical and practical implications. Theoretically, they highlight the complex interplay between emotional regulation strategies, cognitive biases, and RESE, reinforcing and extending Gross’s Emotion Regulation Process Model (Gross, 1998) by elucidating how cognitive-emotional interactions contribute to depression. Specifically, while Gross’s model emphasizes how emotion regulation strategies (e.g., expressive suppression) can influence emotional outcomes, our findings suggest that cognitive processes, such as negative attentional bias, may serve as critical mediators in this process. This cognitive-emotional interaction underscores the need for more integrative models of emotion regulation, which consider not only the strategies individuals use to regulate emotions but also how these strategies may interact with cognitive vulnerabilities to influence mental health outcomes. Our study extends Gross’s model by illustrating that the effectiveness of emotion regulation strategies like expressive suppression may depend on individual differences in emotional self-efficacy and cognitive biases, leading to varying outcomes in terms of depressive symptoms. Moreover, the current findings contribute to the cognitive vulnerability model of depression (Beck, 2008; Alloy and Abramson, 1988), which posits that cognitive biases, such as negative attentional bias, are central to the development and persistence of depressive symptoms. By highlighting how expressive suppression enhances negative attentional bias, our study provides empirical support for the idea that emotion regulation strategies might not only influence emotional responses but also shape the way individuals process information. This suggests that emotion regulation and cognitive biases should be viewed as interconnected rather than isolated processes, which could lead to a deeper understanding of the cognitive-emotional feedback loops that sustain depression. Therefore, our findings call for an expansion of cognitive vulnerability models to include emotional regulation strategies as key components that interact with cognitive biases to influence depression outcomes. Practically, identifying negative attentional bias as a mediator and RESE as a moderator enriches understanding of vulnerability to depression among college students, offering novel insights for tailored interventions and preventative strategies. Importantly, one practical implication of these findings is the potential for developing interventions aimed at enhancing RESE (Hervás and Jódar, 2013). Based on our results, such interventions could be operationalized by combining cognitive training and self-efficacy reinforcement. Cognitive training could focus on helping individuals recognize and modify negative attentional biases through strategies like cognitive-behavioral techniques, mindfulness exercises, and attentional bias modification (Lodder and van der Veen, 2020). Meanwhile, self-efficacy reinforcement could involve structured activities designed to increase confidence in one’s ability to manage emotional experiences, such as emotion regulation skills training, self-reflection exercises, and goal-setting to track emotional regulation progress (Gross, 2015). To further enhance the effectiveness of these interventions, a stepped framework could be employed (Ludwig and Lutz, 2017). The first step would involve assessing individuals’ baseline levels of RESE and cognitive biases. The second step would focus on providing psychoeducation on emotion regulation and the impact of cognitive biases on depression, followed by targeted exercises to modify negative attentional bias. The third step would introduce self-efficacy reinforcement strategies, gradually increasing the complexity of emotion regulation tasks as individuals demonstrate progress. This stepped approach allows for personalized interventions that can be adapted based on the individual’s progress, ensuring that each participant receives the appropriate level of support. In summary, operationalizing RESE enhancement interventions through a combination of cognitive training and self-efficacy reinforcement, within a stepped framework, can offer a structured and individualized approach to addressing vulnerability to depression, particularly among college students.

## 5 Conclusion

This study explored a moderated mediation model among college students, demonstrating that negative attentional bias mediates the relationship between ES and subthreshold depression. Furthermore, RESE moderated both the direct and indirect relationships, with higher RESE attenuating these associations. Overall, the study underscores the critical roles of cognitive biases and emotional self-efficacy in the pathway linking expressive suppression and depression, providing valuable direction for developing targeted interventions and preventive measures tailored to college students.

## Data Availability

The original contributions presented in this study are included in this article/supplementary material, further inquiries can be directed to the corresponding author.
